# Geographic variation in gene flow from a genetically distinct migratory ecotype drives population genetic structure of coastal Atlantic cod (*Gadus morhua* L.)

**DOI:** 10.1111/eva.13422

**Published:** 2022-06-21

**Authors:** Bjoerghild Breistein, Geir Dahle, Torild Johansen, Francois Besnier, Maria Quintela, Per Erik Jorde, Halvor Knutsen, Jon‐Ivar Westgaard, Kjell Nedreaas, Eva Farestveit, Kevin Alan Glover

**Affiliations:** ^1^ Institute of Marine Research Bergen Norway; ^2^ Department of Biology University of Bergen Bergen Norway; ^3^ Institute of Marine Research Tromsø Norway; ^4^ Institute of Marine Research Flødevigen Norway; ^5^ Centre for Coastal Research, Department of Natural Sciences University of Agder Kristiansand Norway

**Keywords:** fishery, gene‐ flow, genome, haplotype, introgression, north East Arctic cod, North Sea cod, Norwegian coastal cod

## Abstract

Identifying how physical and biotic factors shape genetic connectivity among populations in time and space is essential to our understanding of the evolutionary trajectory as well as the management of marine species. Atlantic cod is a widespread and commercially important marine species displaying several ecotypes with different life history strategies. Using three sets of SNPs: neutral, informative, and genome‐inversion linked, we studied population genetic structure of ~2500 coastal Atlantic cod (CC) from 40 locations along Norway's 2500 km coastline, including nine fjords. We observed: (1) a genetic cline, suggesting a mechanism of isolation by distance, characterized by a declining *F*_ST_ between CC and North East Arctic Cod (NEAC—genetically distinct migratory ecotype) with increasing latitude, (2) that in the north, samples of CC from outer‐fjord areas were genetically more similar to NEAC than were samples of CC from their corresponding inner‐fjord areas, (3) greater population genetic differentiation among CC sampled from outer‐fjord areas along the coast, than among CC sampled from their corresponding inner‐fjord areas, (4) genetic differentiation among samples of CC from both within and among fjords. Collectively, these results permit us to draw two main conclusions. First, that differences in the relative presence of the genetically highly distinct, migratory ecotype NEAC, declining from north to south and from outer to inner fjord, plays the major role in driving population genetic structure of the Norwegian CC. Second, that there is limited connectivity between CC from different fjords. These results suggest that the current management units implemented for this species in Norway should be divided into smaller entities. Furthermore, the situation where introgression from one ecotype drives population genetic structure of another, as is the case here, may exist in other species and geographical regions, thus creating additional challenges for sustainable fisheries management.

## 1 INTRODUCTION

Identifying how the physical environment and biological processes combine to shape genetic connectivity among populations in time and space is essential to our understanding of how ecosystems and species function. In addition, it provides us with essential knowledge which is required to base informed decisions for sustainable management, and where relevant, exploitation (Reiss et al., 2009; Waples et al., 2008).

Species inhibiting terrestrial environments as well as freshwater ecosystems often show distinct population genetic structure, leading to a strongly supported management of stocks and management units (Palsbøll et al., 2007). In such environments, barriers to migration and therefore gene‐flow are typically easily identified. Both biotic (e.g., competition and predation), abiotic (e.g., soil type and topography) factors (Ibáñez et al., 2006; Reiss et al., 2009), as well as man‐made structures affect gene‐flow (Harris et al., 2009; Seidler et al., 2015).

In the ocean, and in stark contrast to terrestrial and freshwater systems, barriers limiting dispersal and gene‐flow are often more subtle or cryptic to human perception. In addition, even highly distinct marine fish populations may periodically overlap thus complicating the picture of genetic and physical separation (Berg et al., 2020; Dahle, Johansen, et al., 2018; Johansen et al., 2018; Michalsen et al., 2014). A range of factors influence genetic isolation among marine fish populations, including historical factors (Mattingsdal et al., 2020; Quintela et al., 2020), environmental gradients (Johannesson et al., 2020; Patarnello et al., 2007; Reid et al., 2017), bathymetric boundaries (Catarino et al., 2015, 2017; Knutsen et al., 2009), life‐history variants including ecotypes (Kirubakaran et al., 2016; Michalsen et al., 2014), sex‐dependent philopatry (Ashe et al., 2015), artificial construction of novel marine habitats (Quintela et al., 2021), and physical distance (Dahle, Johansen, et al., 2018; Drinan et al., 2018; Pogson et al., 2001). Many marine fish populations are also typically very large, and thus display limited genetic drift and correspondingly slow rates of divergence at neutral markers (Han et al., 2020). Consequently, genetic methods involving a handful of random and typically selectively neutral genetic markers, such as microsatellites, often struggle to resolve population structure (Jorde, Synnes, et al., 2018; Ryman et al., 2006). However, the proliferation of genomic methods and their application is now revealing previously hidden levels of genetic and genomic diversity among marine fish populations, including examples as diverse as Ballan wrasse (*Labrus bergylta*) (Jansson et al., 2020), Atlantic cod (*Gadus morhua*) (Kirubakaran et al., 2016; Sodeland et al., 2016), European sprat (*Sprattus sprattus*) (Quintela et al., 2020), and Atlantic herring (*Clupea harengus*) (Han et al., 2020; Martinez Barrio et al., 2016). Therefore, genomics, or the application of carefully selected panels of informative markers that have been mined from the genome, now provide unprecedented opportunities to study the evolutionary relationships among marine fish populations, and unravel their underlying causative mechanisms.

The Atlantic cod is an economically important demersal fish distributed throughout the North Atlantic. Historically, this species has formed the basis of important fisheries in many countries, some of which have had well‐documented stock collapses (Myers et al., 1997; Pershing et al., 2015). Partly due to its economic and cultural significance, this species has been given considerable attention in the scientific community, and a well characterized genome and associated resources are therefore available (Kirubakaran et al., 2020; Star et al., 2011; Tørresen et al., 2017). Cod is characterized by genetically distinct populations displaying different life history strategies and habitat preferences. Many of these ecotypes are widespread throughout the species range, and there is also evidence of parallel evolution between highly migratory and more stationary ecotypes on both sides of the Atlantic (Bradbury et al., 2010; Sinclair‐Waters et al., 2018). The waters of Canada, Greenland, Iceland, and Norway all contain ecotypes or groups of cod with different migrating patterns (Morris et al., 2003; Pampoulie et al., 2008; Storr‐Paulsen et al., 2004), as well as populations preferring different habitats regarding elements like depth and temperature (Berg et al., 2016; Kirubakaran et al., 2016; Pampoulie et al., 2008; Sodeland et al., 2016).

Norway is home to some of the most numerous cod populations and fisheries remaining in the Atlantic (Bergstad et al., 1987; Dahle, Quintela, et al., 2018). Previous genetic studies, using hemoglobin (Dahle & Jorstad, 1993), mtDNA (Dahle, 1991), microsatellites (Dahle, Quintela, et al., 2018; Glover et al., 2011; Jorde et al., 2007; Knutsen et al., 2003; Knutsen et al., 2011), *Pan*I (Fevolden & Pogson, 1997; Sarvas & Fevolden, 2005; Skarstein et al., 2007), and genome‐distributed SNPs (Berg et al., 2016; Johansen et al., 2020; Moen et al., 2008; Sodeland et al., 2016), have given insights into population structure and connectivity within this region. These studies have found notable genetic differences in parts of the genome (including genomic inversions) between the migratory North East Arctic Cod (NEAC) and Norwegian coastal cod (CC; Berg et al., 2016; Kirubakaran et al., 2016). They have also revealed genetic differentiation among CC populations in a north‐to‐south gradient (Dahle, Quintela, et al., 2018; Johansen et al., 2020). This has been suggested to be influenced by a geographic gradient of interbreeding between the genetically highly distinct NEAC and CC toward the north (Dahle, Quintela, et al., 2018), and CC and North Sea cod (NSC) toward the south (Jorde et al., 2021).

**FIGURE 1:**
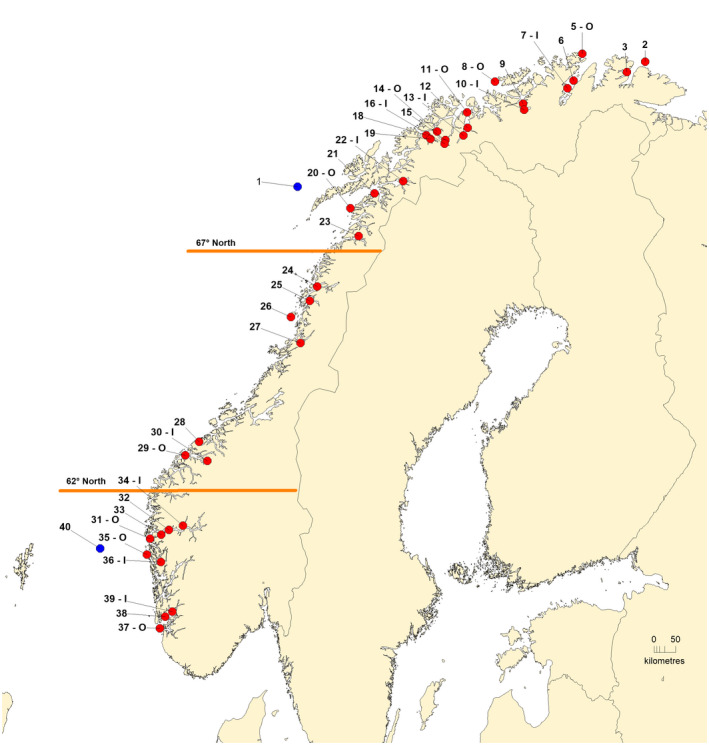
Map of sampling sites with CC (red dots) used in study. NEAC (1) and NSC (40) reference samples shown as blue dots. Numbers correspond to sampling location ID (Table 2), the stations with—I and—O are used in the inner vs. outer comparisons. Selected latitudes marked in orange

The Norwegian coastline is approximately 2500 km long, consisting of multiple fjords of varying length (Figure 1). This seascape offers substantial potential for the development of genetically isolated populations of cod, especially within fjords; for example, both presence (Dahle, Johansen, et al., 2018; Johansen et al., 2018) and possibly interbreeding (Dahle, Quintela, et al., 2018) between NEAC and CC varies according to the seascape. In southern Norway, genetic studies of cod along the Skagerrak coast have revealed two genetically and biologically distinct ecotypes that partly mix on the coast, one being more common inside fjords, while the other one seems to occur in higher frequencies in exposed areas, belonging to the North Sea component (Jorde, Kleiven, et al., 2018; Knutsen et al., 2018; Sodeland et al., 2016). The NSC components are suggested to have dispersed from either the North Sea or from offshore areas of Skagerrak (Barth et al., 2019; Knutsen et al., 2004) In addition, there is evidence for weak but significant population structure along Skagerrak (Barth et al., 2019; Jorde et al., 2007; Knutsen et al., 2003; Wenne et al., 2020). In northern Norway however, where we find most of the Norwegian CC, analysis of population structure within and among fjords has been studied in far less detail (Skarstein et al., 2007), and there is a significant gap in knowledge from this ecologically and economically important region for cod.

The potential to overharvest and thereafter deplete fish stocks in the world's oceans through misinformed and/or poor management regimes represent a persistent global problem. Therefore, a thorough understanding of population genetic structure represent an important component for sustainable management (Reiss et al., 2009; Waples et al., 2008). Given its commercial importance throughout large parts of the north Atlantic, its well‐documented history of over‐exploitation, and the availability of genomic resources, the Atlantic cod serves as a good case study for further investigation of the processes and mechanisms underpinning a species with a complicated population genetic structure. Therefore in this study, we provide an in‐depth analysis of the population genetic structure of approximately 2500 CC collected from 40 locations in Norway, focusing on outer versus inner‐fjord transects in a N‐S latitudinal gradient. In order to study this, three novel panels of SNPs were carefully selected from the genome, including those that: (i) have been demonstrated to be selectively neutral, (ii) are latitude‐informative, and (iii) are linked to genome‐inversions and under selection in this geographic region.

## 2 MATERIALS AND METHODS

### 2.1 Samples

This study is based on the analysis of 2768 cod sampled from 40 locations in Norway, including to two samples of NEAC and NSC for reference (Figure 1, Table 1). Samples were collected primarily from mature adult fish on their spawning grounds in the following four ways: scientific research cruises conducted by the Institute of Marine Research in Norway, monitoring surveys conducted by the Norwegian Directorate of Fisheries, sampling cod from commercial catches landed by local fishermen, and collaboration with other projects where cod samples were made available. From each fish, biological data including otoliths were collected when possible. Otoliths were used to phenotypically classify fish as NEAC (otolith categories 5 = certain or 4 = uncertain NEAC), or CC (otolith categories 1 = certain or 2 = uncertain CC), according to (Berg & Albert, 2003; Rollefsen, 1933). The NSC otolith is classified as category 1 (or 2) and thus not distinguishable from CC. All fish were genotyped irrespective of otolith category, although fish belonging to categories 4 and 5 were removed from the statistical analyses. A subset of the data, containing 1170 individuals from the 9 fjords for which both inner and outer samples were available, were used in some of the analyses, as reported the data analysis section.

**TABLE 1:** Summary of information on sampling sites

Sites	Short name	ID	Sampling date	*N*	NEAC and missing otholite	% CC	*N* after purging	Markers out of HWE	Hobs	Hexp
NEAC	NEAC	1	apr.03	66	66	0	64	1	0.305	0.315
Tanafjorden	Tana_O	2	feb.19	50	32	36	18	1	0.314	0.324
Tanafjorden	Tana_M	3	feb.19	188	54	71.3	127	7	0.299	0.321
Tanafjorden	Tana_I	4	oct.19	8	3	62.5	NA	NA	NA	NA
Porsangerfjorden	Pors_O	5	feb.19	144	87	39.6	57	4	0.3	0.323
Porsangerfjorden	Pors_M	6	apr.19	158	33	79.1	124	7	0.304	0.327
Porsangerfjorden	Pors_I	7	feb.19	77	12	84.4	65	3	0.301	0.324
Altafjorden	Alta_O	8	feb.19	232	171	26.3	60	2	0.307	0.316
Altafjorden	Alta_M	9	apr.19	124	35	71.8	88	6	0.313	0.331
Altafjorden	Alta_I	10	oct. 19	53	11	79.2	42	0	0.309	0.323
Lyngen	Lyng_O	11	oct. 19	65	10	84.6	51	4	0.293	0.319
Lyngen	Lyng_M	12	mar.19	119	4	96.6	113	4	0.311	0.326
Lyngen	Lyng_I	13	oct. 19	42	7	83.3	35	1	0.314	0.325
Balsfjorden	Bals_O	14	mar.19	70	4	94.3	61	3	0.309	0.328
Balsfjorden	Bals_M	15	oct. 19	66	1	98.5	64	2	0.299	0.325
Balsfjorden	Bals_I	16	apr.19	161	2	98.8	155	10	0.303	0.33
Malangen	Mala_O	17	oct. 19	1	0	100	NA	NA	NA	NA
Malangen	Mala_M	18	oct. 19	23	2	91.3	19	0	0.307	0.318
Malangen	Mala_I	19	oct. 19	37	6	83.8	30	1	0.312	0.325
Vestfjorden	Ofot_O	20	oct. 19	42	6	85.7	34	3	0.302	0.327
Vestfjorden	Ofot_M	21	oct. 19	25	6	76	19	1	0.31	0.326
Ofotfjorden	Ofot_I	22	feb.19	53	3	94.3	49	5	0.31	0.328
Skjærstadfjorden	Skja_M	23	apr.19	47	0	97.9	37	1	0.296	0.317
Ranfjorden	Ranf_O	24	oct. 19	48	0	100	36	1	0.298	0.326
Vefsnfjorden	Vefs_O	25	oct. 19	27	0	100	25	1	0.307	0.317
Vega	Vega_O	26	mar.19	47	0	100	37	3	0.293	0.323
Bindalsfjorden	Bind_M	27	feb.19	47	1	97.9	27	1	0.298	0.313
Kvernesfjorden	Kver_O	28	mar.19	47	0	100	40	3	0.313	0.320
Midfjorden	Roms_O	29	mar.20	67	1	98.5	61	5	0.296	0.321
Romsdalsfjorden	Roms_I	30	jan.20	47	1	97.9	46	3	0.282	0.314
Sognefjorden	Sogn_O	31	oct. 19	32	0	100	31	1	0.305	0.316
Sognefjorden	Sogn_M	32	jan.20	63	0	100	54	3	0.295	0.32
Sognefjorden	Sogn_M2	33	feb.19	56	0	100	56	1	0.304	0.316
Sognefjorden	Sogn_I	34	sep.19	27	0	100	27	0	0.3	0.313
Øygarden	Hord_O	35	feb.19	100	0	100	91	3	0.29	0.318
Sørfjorden(Osterøy)	Hord_I	36	feb.19	57	0	100	53	3	0.287	0.319
Boknafjorden	Bokn_O	37	nov.19	56	0	100	52	4	0.287	0.314
Boknafjorden	Bokn_M	38	sep.19	56	1	98.2	55	3	0.301	0.326
Boknafjorden	Bokn_I	39	sep.19	58	3	94.8	54	5	0.302	0.325
NSC	NSC	40	mar.07	82	0	0	82	4	0.292	0.312
Total				2768	562		2139	110	0.302	0.321

*Note*: The lettering at the end of the short name indicates whether the station is inner (I), middle (M) or outer (O). *N* is the initial number of samples. “Excluded cod” is NEAC based on otolith type and individuals with unreadable or missing otolith, giving a percentage of secure coastal cod (%CC). *N* after purging are the individuals with acceptable quality. Markers out of HWE, are SNP markers that deviates from Hardy Weinberg equilibrium. Expected Heterozygosity (Hexp) and Observed heterozygosity (Hobs) for SNP markers are also noted.

### 2.2 Choice of SNP's

Three novel and complimentary panels of SNPs were used to genotype all samples in this study. These were selected from the cod genome based upon detailed information from a recent analysis of population genomic diversity of cod in this region (Johansen et al., 2020). We chose a set of 74 genome‐distributed SNPs that were deemed selectively neutral (Johansen et al., 2020) and 48 SNPs from previously documented inversions on linkage groups 1, 2, 7, and 12, and thus likely under strong selection as they tend to segregate between NEAC and CC ecotypes (Berg et al., 2016; Kirubakaran et al., 2016; Sodeland et al., 2016). We added 47 SNPs that have been previously demonstrated to vary with latitude among 5 samples of CC in Norway (Johansen et al., 2020), but are nevertheless located outside the known inversions. For simplicity, the three aforementioned panels will be referred to as *neutral*, *inversion,* and *gradient* panel, respectively. The concept behind these panels of SNPs was to simultaneously capture the neutral and potentially adaptive divergence. SNP assays was created in MassArray Assay Creator, (Agena Bioscience; File S1).

### 2.3 DNA extraction and genotyping

DNA was extracted from the sampled tissues (both fin and gill) with the Qiagen DNA Blood and Tissue kit, following the manufacturer's instructions. Each 96 well plate contained 2 non template controls. DNA from approximately 10% of the individuals was quantified on a NanoDrop 8000, by Thermo Fisher, and DNA diluted according to block wise average. The 2768 individuals were thereafter genotyped in 386‐well format on a Sequenom MassArray platform (Agena Bioscience), as described in (Gabriel et al., 2009).

### 2.4 Data analysis

To ensure reliable analysis, raw data was purged as follows: All cod identified as NEAC by otolith reading, as well as individuals where the otolith could not be reliably read, were excluded from further consideration (Table 1). Furthermore, sampling locations that, based upon otolith reading, were deemed to consist of less than 18 CC were removed completely, except for the reference samples (Table 1). Based upon this procedure, 502 individuals were discarded. Loci with signs of technically poor performance (bad and/or inconstant clustering), as well as those with more than 30% missing data were excluded. This purging resulted in a total of 148 SNP's being scored, representing 65 “neutral” markers, 41 “gradient” markers, and 42 “inversion” markers. A total of 127 individuals showing >25% missing genotypes were also discarded leaving a total of 2139 individuals, including reference samples of NEAC and NSC, in the final dataset for the statistical analysis.

Hardy Weinberg equilibrium tests were performed in *R* (R Development Core Team, 2020) using the package *PopGenReport* (Adamack & Gruber, 2014) with the chi‐square test. Type I errors were corrected for using the Bonferroni correction. Heterozygosity, both expected and observed, was calculated using the R package *adegenet* (Jombart, 2008; Jombart & Ahmed, 2011; Jombart et al., 2010). Allele frequencies were computed with *GenAlEx* (Peakall & Smouse, 2006). The analyses described above were performed on the neutral and the gradient markers. Haplotypes were constructed from individual inversion SNPs with *PHASE* v.2.1.1. (Stephens, 2004; Stephens et al., 2001) and treated as haplotypes or multi‐allelic loci.

To determine potential clustering of individuals based on multilocus genotypes, DAPC plots were produced in *adegenet*, separately for the three groups of markers (i.e., neutral, gradient, and inversion) with 2 principal components plotted (respectively 50, 40, and 70 analyzed to avoid overfitting). In addition, the Bayesian clustering analysis which were performed in *STRUCTURE* v.2.3.4 (Pritchard et al., 2000) on all three sets of markers. The model assuming admixture was used with a length of burn‐in on 100,000 and a run length of 400,000 MCMC, testing for *K* = 1 to *K* = 10 with 3 iterations each. The most likely number of genetic clusters (*K*) were tested for by the means of both the four statistic methods (MedMed, MedMean, MaxMed, and MaxMean) and the ad hoc summary statistic Δ*K* in the Evanno method (Evanno et al., 2005). The plot averaging all 30 runs were produced via *Clumpak* (Kopelman et al., 2015), all implemented in *StructureSelector* (Li & Liu, 2018).

Genetic differentiation among samples was quantified by calculating pairwise *F*_ST_ (Weir & Cockerham, 1984) with *Arlequin* V. 3.5 (Excoffier et al., 2005), testing it with 10,000 permutations, and a significance level of 0.05. The full dataset with 2139 individuals was run separately for each marker group, the reduced, 9‐fjords, dataset of 1170 individuals were split up into “Inner” and “Outer” and run separately for each marker group.

Isolation by distance was tested via Mantel test using *GenAlEx* with 9999 permutations. Shortest distance by water was calculated using the R package *marmap* (Amante & Eakins, 2009; Pante & Simon‐Bouhet, 2013; van Etten, 2017), whereas pairwise *F*_ST_s was obtained from *Arlequin*. Results were visualized in *R* with the package *ggplot2* (Wickham, 2016) plotting *F*_ST_/(1 − *F*_ST_) against geographic distance.

## 3 RESULTS

### 3.1 Data set description

Following genotyping quality checks and removal of NEAC, the main data set consisted of 2139 fish genotyped for 148 markers (Table 1). These included 65 neutral, 41 gradient, and 42 inversion markers (the 42 inversion SNP's were converted subsequently into 5 inversion haplotype markers located on chromosomes 1, 2, 7, and 12).

Expected and observed heterozygosity showed very little variation among samples, whereas the number of markers out of HWE varied between 0 and 10 per sample (out of the 106 SNP markers; Table 1). The percent CC being present in samples prior to purging NEAC according to their otolith classification, ranged from a low of 26.3% in Alta O in the north, to 100% in many of the samples in the west or south of Norway. This clearly demonstrates a higher presence of NEAC in the northern regions.

### 3.2 Overall patterns of genetic differentiation

**FIGURE 2:**
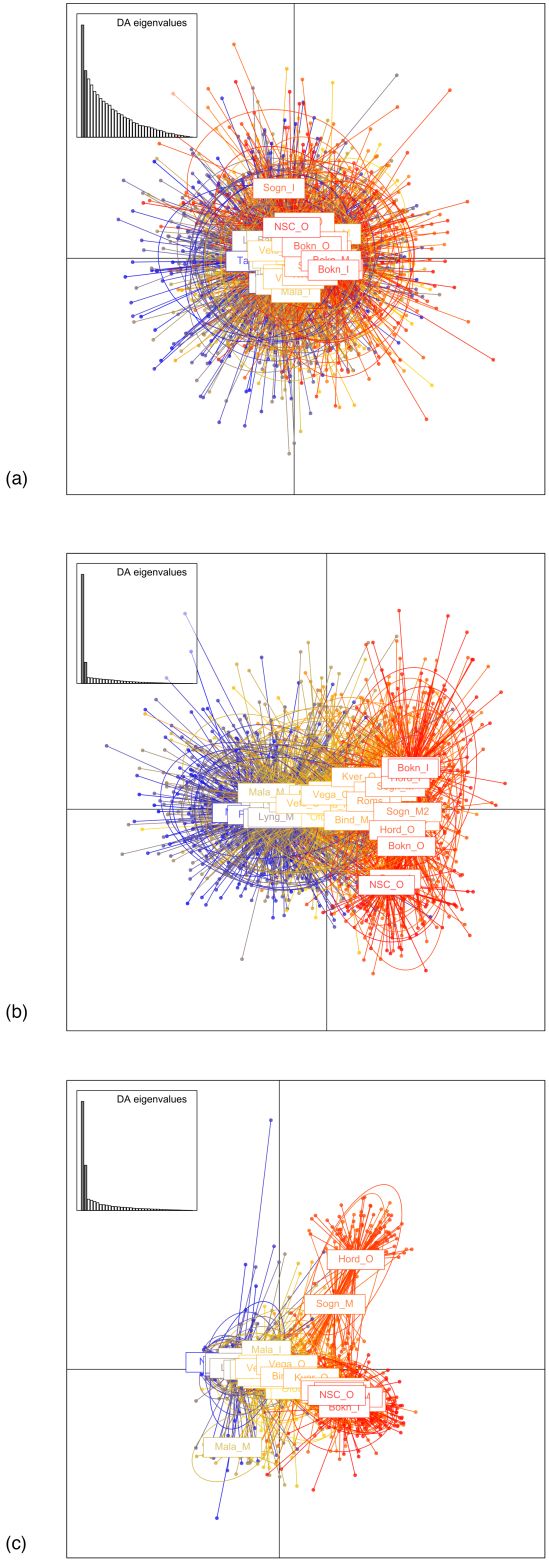
DAPC plots depicting genetic structure among all 40 samples of coastal cod using (a) neutral, (b) gradient, and (c) inversion‐linked SNPs. *X* and *Y* axes determine 13% and 8%, 55% and 10%, and 42% and 17% of the structure seen plots a–c, respectively

Significant genetic variation was observed among the CC samples, although this differed greatly among the sets of markers (Table 2). The largest values were obtained with the inversion markers, followed by the gradient markers. In contrast, the neutral markers displayed very low and statistically nonsignificant global *F*_ST_ values among samples (Table 2). Differences in the degree of differentiation revealed by the three sets of markers was evident in all analyses, as graphically visualized by the DAPC plots (Figure 2). The neutral markers did not resolve any structure while the gradient and inversion markers revealed differentiation primarily along the north–south gradient. As a consequence of the lack of differentiation using the neutral markers, we chose to concentrate the analyses on the gradient and inversion markers in the following work.

**TABLE 2:** Global *F*_ST_ values

	*F*_ST_ CC	*p*‐Value	*F*_ST_ including NEAC/NSC	*p*‐Value
All locations (40)				
Inversions	0.092	<0.001	0.095	<0.001
Gradient SNP's	0.026	<0.001	0.031	<0.001
Neutral SNP's	0.00047	0.14	0.00049	0.11
Inner‐fjord locations (9)				
Inversions	0.090	<0.001	0.10	<0.001
Gradient SNP's	0.023	<0.001	0.029	<0.001
Neutral SNP's	0.00030	0.41	0.0012	0.076
Outer‐fjord locations (9)				
Inversions	0.13	<0.001	0.13	<0.001
Gradient SNP's	0.036	<0.001	0.048	<0.001
Neutral SNP's	0.0010	0.88	−0.00033	0.67

*Note*: Global *F*_ST_ values among three different sets of samples using SNPs from the inversions, gradient‐informative SNPs, and the neutral SNPs.

**FIGURE 3:**
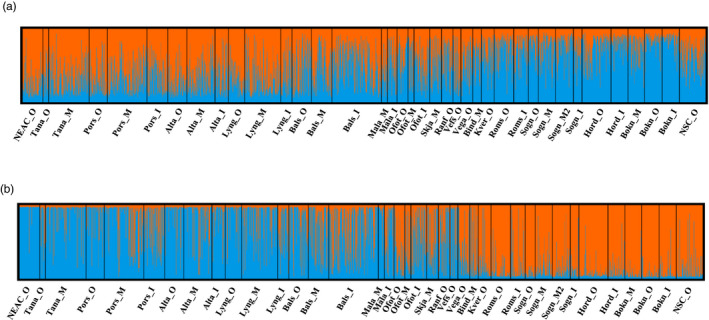
Results of STRUCTURE analysis set at K = 2 using (a) the gradient informative SNPs and (b) SNPs within inversions. K = 2 was selected after visual inspection, given that both K = 2 and 3 were suggested as possibilities by different methods in *StructureSelector*

**FIGURE 4:**
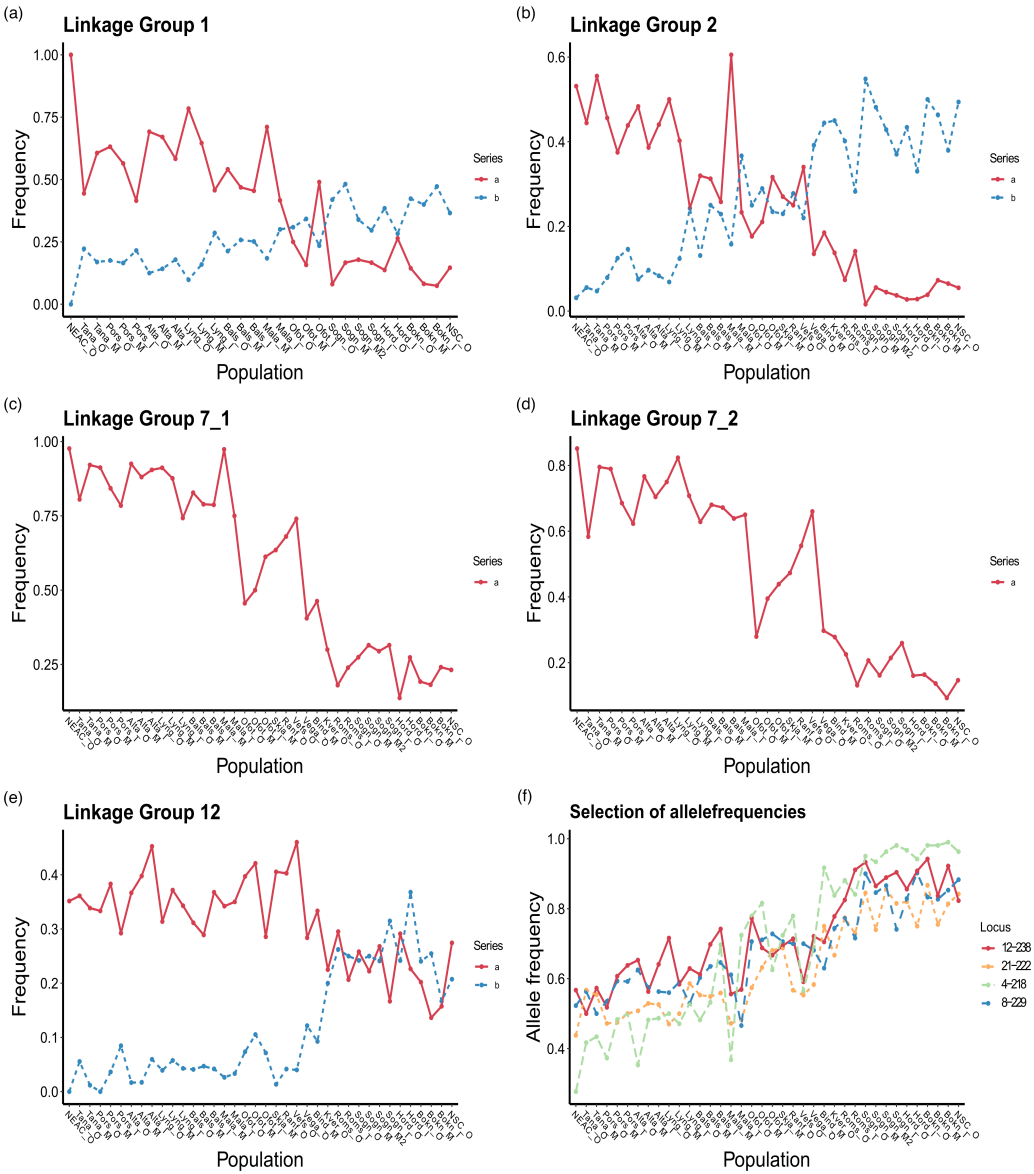
Frequencies of haplotypes that display frequencies of ≥0.4 in any of the samples for (a) linkage group 1, (b) linkage group 2, (c) linkage group 7_1, (d) linkage group 7_2 and (e) linkage group 12. Panel (f) presents the allele frequencies of a selection of loci from the gradient marker panel. Populations are presented from north to south and outer to inner in the graphs

**FIGURE 5:**
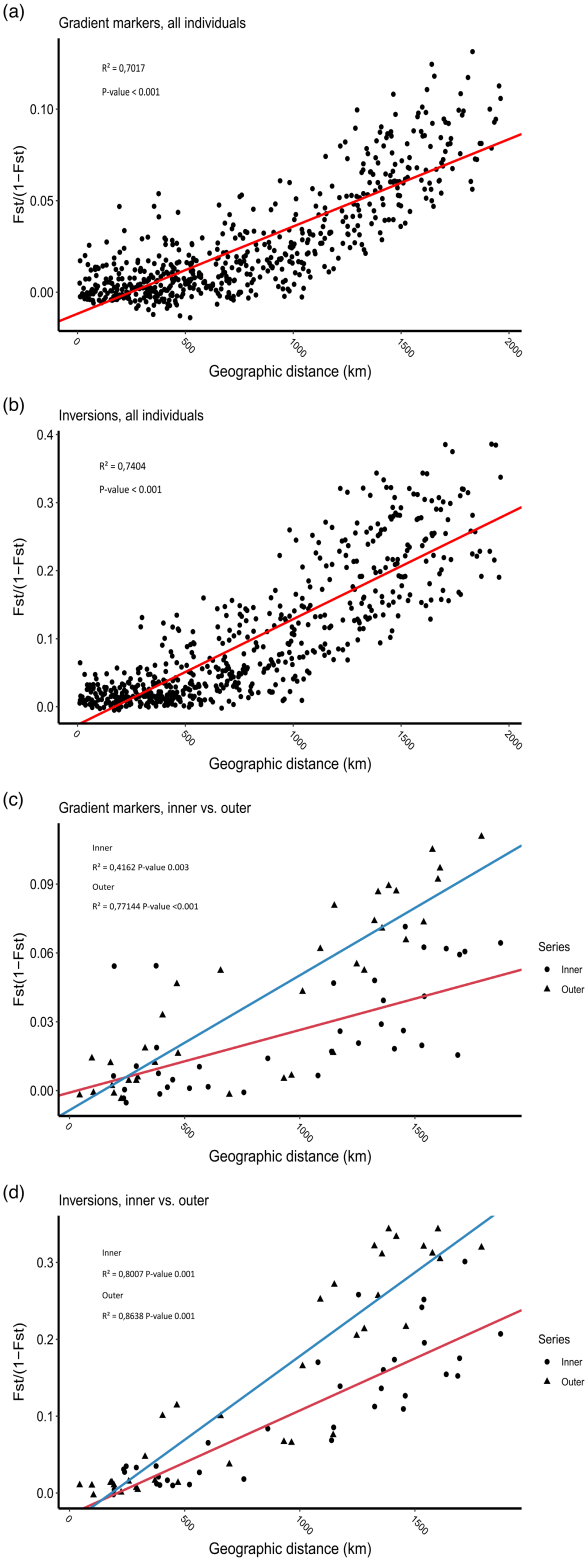
Isolation by distance. Mantel test performed in GenAlEx with 9999 permutations, and *R* ^2^ found to y *R* ^2^ = 0.7017 with a *p* value of 0 for the gradient markers, and *R* ^2^ = 0.7404 with a *p* value < 0.001 for the inversions. In the normalized stations, the gradient markers the inner individuals give *R* ^2^ = 0.4162 with a *p*‐value 0.003, the outer individuals give *R* ^2^ = 0.7714 with a *p*‐value of 0, for the inversions inner individuals *R* ^2^ = 0.8007 with a *p*‐value of 0.001 and outer individuals *R* ^2^ = 0.8638 and *p*‐value 0.001

Structure analysis did not reveal genetically distinct populations, but a gradient of admixture between the two genetic components in the north to south direction for the gradient markers (Figure 3a). The nature of this pattern in population genetic structure was obvious from the spatial allele frequencies of many of the gradient SNPs (Figure 4f), and was also supported by the analysis revealing a strong trend of genetic isolation by distance with all markers (Figure 5a–d). For the inversion markers, which displayed a more abrupt cut‐off, as opposed to gradient in the north to south direction (Figure 3b), a division between the northern component that is genetically most similar to NEAC and the southern component, was observed between Vega at ~65.6°N and Malangen at ~68°N. Between these two regions, individuals belonging to both genetic groups were evident from the inversion markers. Nevertheless, in the region north of Malangen, some of the samples still displayed frequencies of the southern genetic component, and the division was not clear‐cut. The haplotype frequencies of the different inversions support this pattern, where linkage group 1, 2, and 12 showed a gradient, but linkage group 7 indicated a more complex pattern between Malangen and Vega (Figure 4a–e).

Pairwise *F*_ST_ estimates computed among all samples, using both inversion and gradient markers, supported most of the observations from the analyses described above (Figure S3a,b, heat table). That is, larger genetic differences were found among pairs of geographically distant samples than between pairs of samples in close proximity. Nevertheless, the weak indication of a more genetically isolated area from some of the inversions and the *STRUCTURE* plot (Figures 4c,d and 3b) is difficult to discern from the pairwise *F*_ST_ values.

### 3.3 Inner and outer‐fjord samples

The data set included nine fjords that contained both an inner (I) and paired outer‐fjord (O) sample (Figure 1). Using both the gradient and inversion markers, global *F*_ST_ values were higher among the sub‐set of outer‐fjord samples than among the sub‐set of inner‐fjord samples (Table 2). To illustrate this point, global *F*_ST_ increased from 0.090 among the inner‐fjord to 0.13 among the outer‐fjord samples, and from 0.023 among the inner‐fjord samples to 0.036 among the outer‐fjord samples, using the inversion and gradient markers, respectively.

**FIGURE 6:**
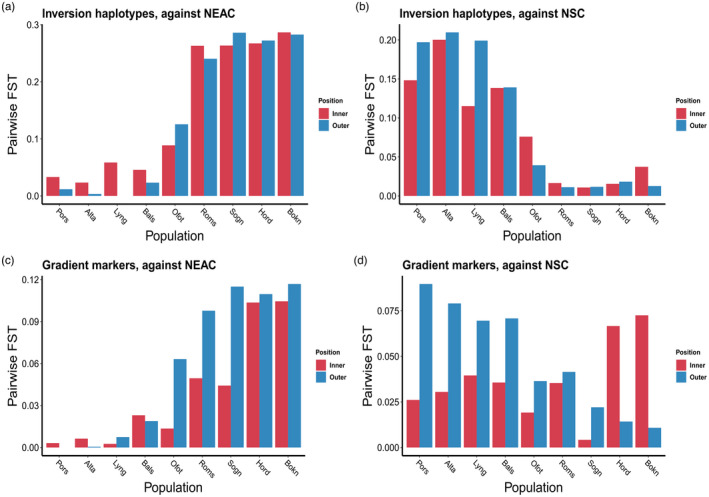
Pairwise *F*_ST_ values between CC samples and the reference samples of NEAC. The analysis was only performed on pairs of inner and corresponding outer‐fjord samples

Looking specifically at the genetic relationship between samples of CC and the reference sample of NEAC, evidence of an inner‐outer cline (Figure 6a,b), in some ways similar to the north–south cline described in detail above, was observed. Looking first at the inversion markers within fjords, this pattern was well illustrated for example in Porsangerfjorden in the north, where the pairwise *F*_ST_ between the inner sample and NEAC was 0.033, but only 0.012 between the outer sample and NEAC (Figure 6b). Likewise, the pairwise *F*_ST_ between the inner sample in Alta and NEAC was 0.023, but 0.003 between the outer sample and NEAC (Figure 6b). This pattern was less distinct in the gradient markers (Figure 6a). In contrast to the above‐mentioned patterns seen in the northern samples (Figure 6a,b), samples collected from south of Balsfjorden displayed less distinction between the inner and outer‐fjord samples in their similarity to NEAC.

When comparing the CC samples with NSC reference sample, the above‐mentioned trends between outer and inner‐CC samples and NEAC reference samples were to a degree inverted (Figure 6c,d). Outer‐fjord samples in Northern Norway displayed a greater genetic distance to NSC than inner‐fjord samples. Southern fjord samples had weak indications of varying relationships between inner and outer‐fjord samples to NSC (Figure 6c,d). For example, with the gradient markers, both the outer‐fjord samples in the two most southern fjords closest to the North Sea, Hardanger and Boknafjord, were genetically more similar to the NSC than their corresponding inner‐fjord samples (Figure 6c). This was not evident in the inversion markers, but those markers were chosen primarily to separate between NEAC and CC and are thus not necessarily expected to detect any influence of NSC on CC in this southern region.

When comparing the genetic isolation by geographic distance patterns (Figure 5c,d), the outer samples had a higher *R* ^2^ and thus a steeper slope for both the gradient and the inversion markers, indicating that they are more affected by distance than the inner samples.

## 4 DISCUSSION

All populations are subject to the same evolutionary processes that drive the emergence of population genetic structure, for example, gene‐flow, genetic drift, selection, and mutation. However, the physical and biological parameters influencing these processes vary greatly among environments, species, and populations. In turn, these variations create patterns of population genetic structure and connectivity that are unique to each ecosystem and population. In this study, we demonstrate how population genetic structure of coastal cod (CC) along the coast of Norway, including both inner‐outer and north–south gradients, is strongly influenced by introgression from the vastly more abundant and genetically distinct migratory North East Arctic Cod (NEAC) ecotype in the north, and, possibly North Sea Cod (NSC) in the south. We therefore draw two primary conclusions: 1—Introgression of NEAC plays the major role in driving population genetic structure of CC along the long Norwegian coastline, and 2—genetic differentiation among samples of CC from both within and among fjords suggests small‐scale spatial genetic structure. The situation revealed here, that introgression from one genetically distinct ecotype controls population genetic structure of another, may well exist in other species and oceans where ecotypes overlap in time and space. In turn, such complicated scenarios create additional challenges for sustainable management of important fisheries resources.

### 4.1 Introgression from one ecotype drives population genetic structure of the other ecotype

The genomic architecture underpinning differences between highly migratory and more stationary ecotypes of Atlantic cod are primarily restricted to four chromosomal inversions (Berg et al., 2017). The migratory NEAC and the more stationary CC along the Norwegian coast is a well‐studied example of this (Berg et al., 2016; Kirubakaran et al., 2016). Using microsatellites (Dahle, Quintela, et al., 2018), genome‐wide SNPs (Johansen et al., 2020) and sub‐sets of SNPs (Jorde et al., 2021) from the above‐mentioned inversions, previous studies have demonstrated that CC becomes genetically more similar to NEAC toward the north of Norway. However, genetic similarity observed between the northern most CC samples and NEAC in our study are greater than in previous studies. This difference likely stems from the use of different sets of markers, and/or the fact that in this study, we sampled further into the north of Norway where there is even greater presence of NEAC. In addition, the most recent of these studies concluded that the cline reflects introgression of NEAC in CC populations and not simply physical mixture, and also that CC merges with NSC toward the south (Jorde et al., 2021). Our results from an extensive analysis with multiple sets of SNPs both confirm the results of earlier studies but also expand knowledge due to more extensive sampling, numbers of markers used, and a unique matched inner and outer‐fjord sampling regime for the first time.

We detected greater population genetic differentiation among the outer‐fjord samples of CC along the coast, than among their corresponding inner‐fjord CC samples. This result may appear paradoxical because there are larger physical distances between the inner‐fjord samples (therefore less opportunity for gene‐flow), there is greater potential for physically isolated populations in inner‐fjord areas (smaller populations and more potential for genetic drift), and the inner‐fjord habitats are likely to be more divergent (more potential for divergent selection and adaptation). However, when combined with the fact that the outer‐fjord samples were genetically more similar to NEAC than their corresponding inner‐fjord sample, it is clear that as for the north–south gradient already discussed, this pattern of gene‐flow appears to be the major driver of the observed genetic differences among CC populations. South of 62°N, where NEAC does not seem to influence the structure, there are indications that NSC, as described in Jorde et al., 2021, is a driving element in population structuring. Both NSC and CC are present on the outer‐coast areas, and it is therefore reasonable to assume that they will affect CC in the outer areas greater than CC from the inner parts of the same fjord. More specific markers are needed to determine the level of the influence of NSC in the south, however.

Coexisting ecotypes of cod are commonly found in several parts of the species range. For example, in Iceland, two distinct cod ecotypes overlap in time and space, yet differ in their migrating behavior, where one performs deep water feeding migrations (Grabowski et al., 2011; Pampoulie et al., 2006). In addition, in Atlantic Canada, cod are separated into the southern and northern stocks, varying both in temperature preference and migratory behavior (Bradbury et al., 2010, 2013; Sinclair‐Waters et al., 2018). Finally, in Greenland, the fjord cod type deviates from the coastal type by being more stationary (Storr‐Paulsen et al., 2004). These examples of migratory vs nonmigratory ecotypes throughout different locations in the north Atlantic are similar to the situation observed along the coast of Norway, with the migratory NEAC influencing the more stationary CC.

Ecotypes and morphotypes are found in a multitude of species. One example of this is the European anchovy (*Engraulis encrasicolus*), where the 2016 Le Moan study showed parallel genetic divergence between pairs of ecotypes based on coastal or marine habitat, on both sides of the Iberian Peninsula. It also showed that these ecotypes hybridize and backcross, complicating management guidelines further (Le Moan et al., 2016). Another example can be found on the coast of Spain, where two genetically distinct Ballan wrasse morphotypes displaying different spotting and life‐history patterns overlap in time and space. However, both the phenotypic and genetic differences between these ballan wrasse morphotypes diminishes to the north (Casas et al., 2021; Quintela et al., 2016), once again complicating management regimes. Thus, our findings of the genetic structure of Norwegian CC being driven by a different ecotype has wider implications for other species and locations where different ecotypes coexist.

### 4.2 Small‐scale spatial genetic structure

The observed genetic difference between pairs of samples of CC from inner‐ and outer‐fjord areas, and between adjacent inner fjords, especially in the north of Norway, strongly suggests limited genetic connectivity among CC from separate fjords. Although as detailed above, these differences primarily arise through the patterns of interbreeding between NEAC and CC. If there was substantial gene‐flow between CC from inner‐ and outer‐fjord areas, and among fjords, then the geographic pattern of NEAC interbreeding would most likely be eroded or cancelled, as already concluded for the north–south pattern observed in this region (Dahle, Quintela, et al., 2018; Johansen et al., 2020; Jorde et al., 2021). Therefore, our data demonstrate that there are small‐scale spatial genetic differences among CC in this region. The nature of fjords, where some are narrow and have a low water exchange with the open ocean, decreases the amount of eggs and larvae that can disperse in and out of the fjord, and thus in turn, may limit gene‐flow with the surrounding areas (Bergstad et al., 2008; Ciannelli et al., 2010; Jung et al., 2012; Knutsen et al., 2007; Rogers et al., 2014). This is consistent with our observations here, and it is likely that such mechanisms are at work. Within‐fjord population genetic structure has also been observed in southern Norway (58° North), with similar conclusions to the present study of limited connectivity (Barth et al., 2019; Knutsen et al., 2011).

Small‐scale spatial genetic structure has also been observed among samples of cod from environments other than fjords. For example, a recent study in the Baltic Sea reported sub‐structuring in samples from the West Baltic stock, indicating an isolation by distance pattern, most likely driven by salinity (Wenne et al., 2020). Furthermore, on the east coast of Canada, cod from Gilbert bay were found to belong to a different genetic component than the surrounding stocks, possibly connected to temperature tolerance (Ruzzante et al., 2000). In addition, a study revealed that analyzing Canadian stocks on genetic islands of divergence instead of by neutral SNP's, reveals population structure not tightly linked to geographic features (Bradbury et al., 2013).

### 4.3 Management implications for cod and other marine fish stocks

Throughout its natural distribution, many Atlantic cod stocks have declined, with well‐profiled examples of fishery collapses in some regions (Myers et al., 1997; Pershing et al., 2015). Norway is home to the largest remaining sustainable Atlantic cod fishery in the north Atlantic (Dahle, Quintela, et al., 2018), with known challenges for fishery management due to the interactions between the numerically abundant NEAC and more local CC stocks that overlap on spawning grounds (Dahle, Johansen, et al., 2018; Johansen et al., 2018). Similar situations and thus management challenges are seen throughout the distribution of this species. For example, in the sea surrounding Iceland, where two genetically distinct ecotypes overlap in time and space (Pálsson & Thorsteinsson, 2003; Pampoulie et al., 2006), they are still only regarded as a single management unit (Romito et al., 2019). Also, several studies on the east coast of Canada have identified a complex pattern in the population genetic structure exceeding the then 6 management units in that region (Bradbury et al., 2010, 2013, 2014; COSEWIC, 2010). In Norway, current management regimes manage CC as three stocks (ICES, 2021). However, our results suggest limited connectivity between CC from adjacent fjords, and therefore suggest that an increase in the number of management units is required. Therefore, both in Norway and on a broader scale, the accuracy of the scientific advice for this species still needs further revision, but just as importantly, the regulatory authorities also need to absorb this new information and adjust management and harvest regimes accordingly. Finally, the case revealed here, that introgression from a genetically distinct ecotype controls population genetic of another, adds additional complications to delineating, and elucidating management boundaries and regimes. This situation is not unique to Atlantic cod and may well be the case for many other marine species throughout the world's oceans.

## CONFLICT OF INTEREST

None declared.

## Supporting information


Appendix S1.

## Data Availability

The data that support the findings of this study are to be deposited in the electronic archive at Institute of Marine Research: https://hdl.handle.net/11250/2996961 upon publication of this manuscript. It consists of one excel file with 3 sheets, one sheet per marker group for all viable individuals.
