# Supplementary material for: Geographic variation in gene flow from a genetically distinct migratory ecotype drives population genetic structure of coastal Atlantic cod (Gadus morhua L.)
Source: Evol Appl. 2022 Jun 21;15(7):1162–76. doi: 10.1111/eva.13422 (PMC9309456; doi:10.1111/eva.13422)
Supplement: Supplementary file 1 — Appendix S1. [file EVA-15-1162-s001.pdf]

Supplementary materials

Assay	SNP_ID	2nd-PCRP	1st-PCRP	UEP_SEQ	EXT1 CALL
W1	8-66	ACGTTGGATGTAAACCTTCACCTACTCCCC	ACGTTGGATGGATTCATTCTGAGAAGAGCC	CTACTCCCCCCATTTTG	C
W1	7-52	ACGTTGGATGTGTGTTAACAGGACTGCTGG	ACGTTGGATGTGGTAATCATCCCCACTTGC	ATCAGATGTCAACCCAC	A
W1	14-201	ACGTTGGATGAAGTGTTGTAGTGGTCGGTG	ACGTTGGATGACCATGAAGAGGTTCATGGC	GCACAAAGGTCACGAAG	C
W1	8-194	ACGTTGGATGCGAGGCTGCTTAGTCATTTG	ACGTTGGATGACGACGCCAGGATTTATCTC	CGCTGCAAATCTCGCTAA	G
W1	8-60	ACGTTGGATGAAGCAAAGTGCAGCCTTTCC	ACGTTGGATGGGAAAATGCTGCACAATAGG	tGGTACACACTACAGGAG	T
W1	1-13	ACGTTGGATGGTGACTTAGGTCGAGAAACG	ACGTTGGATGATTGCTACGGAGGTGTACTG	gAAGGGATGTCATTGAGT	C
W1	1-6	ACGTTGGATGCTCTCCCAGAACATACTGAG	ACGTTGGATGTTTGGATCCTGACAGCCTTG	TCTCCCTTGCAATGTGTCA	C
W1	12-75	ACGTTGGATGTTGAGTAGTTAGGACCGTAG	ACGTTGGATGTTTGGGCTGATCTACCAAAG	TTAGGACCGTAGTGTACTG	C
W1	3-81	ACGTTGGATGTCTTGTCTTCGTGTCAGAGG	ACGTTGGATGTGAGCAGTTTACTGTGAGAG	ttTTTCCTCCCTGTTTTGAA	C
W1	8-63	ACGTTGGATGGCGAGAATAGAAGGAAAGGG	ACGTTGGATGTTCAATTGCTGCAGTGGGAC	GGACATATGTCTCTTGATTG	G
W1	11-200	ACGTTGGATGCACCTCACATGGCCTAAATA	ACGTTGGATGAGCTCTGCATGTCTCTAAAC	gGGACAGATAAAACGGTAGA	C
W1	15-147	ACGTTGGATGTAGCATGACGCTACGCTAAG	ACGTTGGATGAAGCGCGTCCATGCTATATG	tGAAGACAAACTATCGCTAAA	G
W1	5-92	ACGTTGGATGCTTTGGCCGTCCTTACAAAC	ACGTTGGATGGGTACAAAGATAATATGCTG	GGCCGTCCTTACAAACTATACC	C
W1	11-129	ACGTTGGATGCTTGCTCCGGCACAAGAAC	ACGTTGGATGTGGTTCTGTGTGGTAGGTTC	ttCTCCAGCCGCTCTGGGACCA	A
W1	16-151	ACGTTGGATGGGCTGTCTTTGTATCCATGC	ACGTTGGATGGATACGTTGGAAGACAGGAC	ggTGTATCCATGCAATACAAAC	A
W1	8-230	ACGTTGGATGTGAGCGTTCAAGGAAGGAGG	ACGTTGGATGGAATCGAACGCAAACTAGGG	AAGGAGGGCTGCGTGTAACGAC	C
W1	7-51	ACGTTGGATGGGCATGTTATGCGCAAACAG	ACGTTGGATGCGAGTCTGCTGCTATGAGTG	AAACAGCAGCCAGCAGTTACATG	A
W1	16-204	ACGTTGGATGTGAAGATAGACAGGAGCTTG	ACGTTGGATGCACACTCACACAGACTACAC	GATAGACAGGAGCTTGTATTTTG	G
W1	11-245	ACGTTGGATGTCTGCTATGGGGTTCATAC	ACGTTGGATGGCAGTGGATATATTCATCAG	TACATTCACATTTAACCTGCTCTG	A
W1	1-11	ACGTTGGATGAATGCCCCTTCTGGAATCTC	ACGTTGGATGATACCAGTAGGATTGCCAGC	GCCCCTTCTGGAATCTCAGTTATT	C
W1	23-185	ACGTTGGATGGGTGCATGCTGCGTAGATAT	ACGTTGGATGGTTGCGGTTTTTTATCCCAG	aaATGCTGCGTAGATATAAGAAAA	C
W1	13-248	ACGTTGGATGTGACGCTACAGAAACACTGG	ACGTTGGATGAGGGAAACAACCGCCTTCAG	ACAGGAGCAGCGTGTTGGAGCTCG	G
W1	18-161	ACGTTGGATGTGATCCGATCCACCTGAACG	ACGTTGGATGATTCCAGATGTTGAGGTCGG	CGCAGACCCTCACAGGGATGTTCGC	C
W1	17-206	ACGTTGGATGACTTTCTCGGTAAAGAGCCC	ACGTTGGATGAAACCAATTGGGTTTGTCGG	CCTCCGCTTCTAACCCTGTTATAATG	A
W1	20-232	ACGTTGGATGTCTACCGAAGTTTTGGAGAC	ACGTTGGATGTCGCTGTCTCATACAGAGTC	GATTAAAATACTATACTGCAACAAAT	C
W1	2-28	ACGTTGGATGGGAGTAGGAGCCACCTATG	ACGTTGGATGCTTCTTCCAGGAGATGAACC	AGGGGAGCCCTTCAGGGGCGCCGCTT	C
W1	1-5	ACGTTGGATGACGAGACGGTTGGTATTGAG	ACGTTGGATGCAAGATTACCCATCTCCCTG	gtGTATTGAGATAGAGACACTGCAAA	T
W1	9-111	ACGTTGGATGTCTATACTACACAGTCACCG	ACGTTGGATGACCATTGTGGACAACCAAAC	TCTATACTACACAGTCACCGTATACTT	C
W1	1-1	ACGTTGGATGTCCATGGAGGGAAAGTGAAG	ACGTTGGATGAGAACGGAATGATGGACCAC	ttATGGAGGGAAAGTGAAGAGTGTTT	C
W2	2-24	ACGTTGGATGGCCAGTAATTAAGCGTGTGC	ACGTTGGATGTGGAAAGCAGAAAAAGTGCG	TTCTACACCATCCTCCA	C
W2	7-53	ACGTTGGATGTTTCTAGCATTACGGCGAGG	ACGTTGGATGATGGTTCCTGGTGTCTGTTG	ATAAACGACCCCACCTC	G
W2	18-162	ACGTTGGATGCTAGGGTTAGGGCTATTCAG	ACGTTGGATGGCATCTTTCATTTGGGTGTAG	GATGTTTTCGGCCAAAG	C
W2	7-30	ACGTTGGATGAGTCCAAGCGCGTGTCCATC	ACGTTGGATGTGAACCTCATCTGGGTAACG	tGCGTGTCCATCTCTGAC	A
W2	5-192	ACGTTGGATGTTCTTCTTCGTCGTGTGTGC	ACGTTGGATGCGCTATGCCTCACAAGAAAC	TGACCGTGGGGCCTATCA	G
W2	7-48	ACGTTGGATGAGCGGCAGGTAAAGGGTTTG	ACGTTGGATGCGGTATACTCCACTTCTGAC	AGGGTTTGGGAATGTGTG	C
W2	7-50	ACGTTGGATGCCTAATGGAATCAGTGCATC	ACGTTGGATGTTTCCTCTGCAGATGGCTTC	TCAGTGCATCCTCTCACCT	T
W2	7-38	ACGTTGGATGTGTTCCGGAAGTCCTGGATG	ACGTTGGATGCAGATCTTGATGTACGTGGC	acGCGGTACGCGTAGATGA	G
W2	2-213	ACGTTGGATGTGGCACGATAGCTCATCATC	ACGTTGGATGTGCAGGTGTCCATAACAGAG	TCCGTAGCTCTTAACGTACA	G
W2	23-221	ACGTTGGATGGCTTCTCACCACAGAATACC	ACGTTGGATGTCACCAGGAATGTCAAGAGG	CAGAATACCACAATTGAACA	C
W2	6-99	ACGTTGGATGTCTTGGCACACTCATCCTTG	ACGTTGGATGCACACTCACTCACAAAGAC	ggGTGTGTGACTGTGCGTTG	G
W2	3-77	ACGTTGGATGCATAAGATAGTCCAAAGCTG	ACGTTGGATGCTCGTCCTCTTTTATGTATTC	tgCCAAAGCTGACAATGTATG	C
W2	2-22	ACGTTGGATGCACAGAACACATCGTTCACC	ACGTTGGATGAATCTATGTGGATGGGAGGG	tCTAGAGCAGATCAGAGATAC	A
W2	19-168	ACGTTGGATGCTGGTGTAAGCCTTACCAAC	ACGTTGGATGTCAGAACATCATTCTGGCCG	atCCTTACCAACTCCAGTGCAT	G
W2	9-112	ACGTTGGATGCGATGTGGAACTTTACATTC	ACGTTGGATGGTTTTGGCTTTAAACAATAT	gTAAGTCCCCAAACTTTACAAC	G
W2	8-65	ACGTTGGATGGTCATTCATTGGTGGCTCTG	ACGTTGGATGGTTACACACGTGATCTCTGG	GGTGGCTCTGAGATTAATAATA	G
W2	22-211	ACGTTGGATGTGACACACCGTAACCATGAC	ACGTTGGATGACTTTACTGTTGGGCAACCG	CCGTAACCATGACGATGACCTGT	G
W2	11-197	ACGTTGGATGAATTTGGTACCGCAGGCTAC	ACGTTGGATGTGTCCATTTGCTTGTTGGTG	aGAAGAAAACACTGAACAAAGAC	T
W2	6-101	ACGTTGGATGACGAGTACGTCTTCCTTTAC	ACGTTGGATGAGGGCTTGCTGTGGTTCTTC	ccGTACGTCTTCCTTTACATCGAC	C
W2	19-234	ACGTTGGATGGTGAATTATGGGCGGGAAAC	ACGTTGGATGCGAATCCTGTCGAACGAAAC	TATGTTCTAGTCTATTGAGTTTAC	G
W2	11-126	ACGTTGGATGTACTTAGGGACTCCGTAGAG	ACGTTGGATGATCCCTGCAACCCGATAAAC	ACTCCGTAGAGAGACGGTCCAGGG	T
W2	4-217	ACGTTGGATGGCTGACTCAGGGTGTATTTC	ACGTTGGATGCTGTCAGAGAGGAGATAAGG	aAGGGTGTATTTCTATGAGAAAAA	C
W2	19-208	ACGTTGGATGCCTTGACGTCAATTGTTGGC	ACGTTGGATGCAGTGGGCACATTTGGAAAG	cTGGCATAGATCGTTCCAGTTTCAT	C
W2	23-186	ACGTTGGATGCATCTATCCACCCAAACACC	ACGTTGGATGAGAACTCTGTAGTCTCAGGC	cCACCCACAGAACGTCCTATGTGCTG	C
W2	2-249	ACGTTGGATGTGCAACACTAGTCCACGATG	ACGTTGGATGGGCGACAAATATCAACACCG	gAACACTAGTCCACGATGGTATAATA	G
W2	9-196	ACGTTGGATGATGGAACAGAAGTATAGCTC	ACGTTGGATGGAGGACATTTTTATGCCGGT	GGAACAGAAGTATAGCTCATTATTTA	G
W2	5-95	ACGTTGGATGTATAAGAGGGCGTCTACTCC	ACGTTGGATGATCCCTAGTCCACAAACAGC	cGCGTCTACTCCATACCTAAAAACTAA	C
W2	19-237	ACGTTGGATGGGTCTTGCGCTATTCTAGTC	ACGTTGGATGTTATCGCAAAACACACCGCC	ACCTTATTTTCTATTGGTTTCTGCCAT	G

W3	4-89	ACGTTGGATGGTATGTCTGTCTGTCCCCGA	ACGTTGGATGTACCAATCATCTGTCCTCTC	CACGTTGCTTCCCACTG	G
W3	7-36	ACGTTGGATGAAGTATGGTCACGTGAGAGG	ACGTTGGATGCAGGAAATTACTACAATCAAG	GCAGTTCCTTTTTACGC	G
W3	13-136	ACGTTGGATGATATAGCCAGCAGAGCAGAG	ACGTTGGATGGTGAAACTCCCTTTGAGCAG	CAGAGCAGAGGAGACGC	A
W3	9-114	ACGTTGGATGGTACAGCATCCTGTTCAAGC	ACGTTGGATGCTAATTGAGCTTTGAGTGGG	AACATGGTAAGTAGCCAC	G
W3	7-55	ACGTTGGATGCATTTGCTCTTCTTCGTCTC	ACGTTGGATGTATGGATCCGAGGAGCATTC	CTTCTTCGTCTCGGCCTCT	C
W3	15-148	ACGTTGGATGTGTGCATCGGTTGGTTCAC	ACGTTGGATGGCAAACTCACACTTGTCCTG	ccGGTCATCATCCGCTTCG	G
W3	17-156	ACGTTGGATGTCAGTTATCTGCTGAGATGG	ACGTTGGATGGCATTACGACACAAATGCAG	aCTGCTGAGATGGACGATA	C
W3	13-138	ACGTTGGATGGTGGATACGGTTCATGTAGG	ACGTTGGATGGGGATTTTCAAACGATGGTG	ggGGTTCATGTAGGCTAGAC	T
W3	10-122	ACGTTGGATGAACTGCTGTGTTTCAACCG	ACGTTGGATGGCGAAAGGTAAACACCTCTG	TTGGTTATGCCGCCACTTAA	G
W3	7-31	ACGTTGGATGTGGTGGAGAAGATGAACGTC	ACGTTGGATGTTTACCGAGTTGCAAAACCC	ctATGAACGTCTTCAAGTCG	A
W3	23-225	ACGTTGGATGCTGTTGACAGAGTTTGCGAG	ACGTTGGATGCTCTCTCTCCTTGGTGTTAG	aAGTTTGCGAGGGTATAGTA	T
W3	22-179	ACGTTGGATGTTCCTGCGGTCCCCGATCTT	ACGTTGGATGTCGTTGTACTTGAGCAGCGG	cCGGTCCCCGATCTTCAAACC	A
W3	6-96	ACGTTGGATGTACTGAAGGTCTGTGTGCTG	ACGTTGGATGTAGTCCACTTGAGACATTAG	GTGCTGCAAAACATGAACAAG	T
W3	15-149	ACGTTGGATGTAATACTGGGTCACTCGCTG	ACGTTGGATGACTTCTCCTGACACTCCTTG	ACTCGCTGATTCTCATTGTCCC	T
W3	11-127	ACGTTGGATGTCTACGGTAGCTCGCAAAAC	ACGTTGGATGTAGCACTTGCGGGTTCAATC	CGCAAAACAAAAGACCAAATTC	C
W3	1-10	ACGTTGGATGCTGGTAGCATGGGTAAATCG	ACGTTGGATGGTCACATGGATACTCTTTGG	tgTGGGTAAATCGGAATAATCT	G
W3	12-71	ACGTTGGATGATGCCTCACATCAAAGTCGC	ACGTTGGATGTGCAACAGAGAGTAAGACAG	CCTCACATCAAAGTCGCATTACA	G
W3	1-2	ACGTTGGATGTACTGGATGGATTCGGTCAC	ACGTTGGATGGGTACAGGCATTTACTCTGA	TGGTGCCAGCAGGATATCAAAAT	C
W3	7-56	ACGTTGGATGCACCCTTTTGCTCAGTGTTG	ACGTTGGATGTGTTCCTGTCTGTATGCGTC	TGTTGGGTGACAAGGATGTAGTC	C
W3	12-250	ACGTTGGATGGAAGCGTTAGAGATGCTGTC	ACGTTGGATGCGCCATCACCAATGATTCTG	CTGCTTTTGCAGATACAGCCCAAA	G
W3	1-4	ACGTTGGATGAAAATGACTGGTGGCAAGGG	ACGTTGGATGATCATCCCTTTTGGAACCCC	gTAACAGTTCATTGATAGGAAGAG	C
W3	7-45	ACGTTGGATGTGCTTCATGTATAACCTGCC	ACGTTGGATGGTGATCAAGTTTGGCATCCG	tCTTCATGTATAACCTGCCAACACT	G
W3	3-214	ACGTTGGATGCCACCACATCTGATACGTTC	ACGTTGGATGGCGTCATGAGGCAGAAGTAG	cgCACATCTGATACGTTCTTCTTCA	A
W3	13-137	ACGTTGGATGGTTCTGTATCACACACCTTC	ACGTTGGATGTGCTTCTCTGTCTCAGGTTC	gTGTAGTATCCAATAGCAAAATTTC	G
W3	3-239	ACGTTGGATGCATTCCATCTCCAGGTTGGC	ACGTTGGATGATAGATCGCTGCTGATGGAG	atCTCGTGATGCTGAACAGAACCCCT	C
W3	16-154	ACGTTGGATGGGCTGGATATAGATAGACAC	ACGTTGGATGCCTGTGTTTATCTTTAGGCTC	GCTGGATATAGATAGACACTAGACAT	C
W3	22-210	ACGTTGGATGTTTTCCACGGTGCCTTTCTC	ACGTTGGATGAGGTTGTTGTTGTCGTAGGG	ctACGGTGCCTTTCTCATTCTATCTAC	A
W3	7-58	ACGTTGGATGAAGATTGGCAGTCTCTTGGC	ACGTTGGATGGCAAGTATCAAGTTGGGCAG	ttaTGTCAAATATGTATTTTTTTTTCC	T
W4	19-233	ACGTTGGATGTGTTGTTCTGCAGGGCAAAC	ACGTTGGATGCTCTCCTCTTGCAGTACTTC	CACCATGGCCAGCACCG	C
W4	16-155	ACGTTGGATGATTTCCTGTCACACGAGTGC	ACGTTGGATGACATGTTTTCCATAGGCCCC	CACGAGTGCGGGAGTCA	C
W4	10-115	ACGTTGGATGGCCAAAGCAGCACATAACTC	ACGTTGGATGTCTTTGCGCAGGAAGTGTTG	tTGCCTTGCCTTTGCTAC	A
W4	1-7	ACGTTGGATGTGGGAACTTTTTCAGCACAG	ACGTTGGATGCGCATCTCCAGGTCTTTAG	TTCCTGAAAAAAAGCCTTG	C
W4	21-222	ACGTTGGATGTTGTCTCCTGTTTCGCTACG	ACGTTGGATGACGTAACCGACAGTACGTCC	GATATCTCCGATGCTAGG	G
W4	20-171	ACGTTGGATGCCAAGAGAACCTGGAGGAAG	ACGTTGGATGCCATTTCCTTACCTAGAGGC	GGGCAGAGGTTTAATGAG	G
W4	7-39	ACGTTGGATGTCTTCCTCATGAGGTTGGTG	ACGTTGGATGTCACTCCTCATTCACTCTCC	AGGTTGGTGGGCTTCAGGT	G
W4	12-68	ACGTTGGATGACACCGTGAAGCAGGCTTTG	ACGTTGGATGTCACCAACAGTGCAAATGCG	aAGGCTTTGGGAGATGTGT	T
W4	8-229	ACGTTGGATGTCGGGTTCCAATCGGATAAC	ACGTTGGATGAGGAAAGACATTCGGATCGG	TCCAATCGGATAACTACCCT	A
W4	14-141	ACGTTGGATGGGGCATCTGAAGAACCTAAC	ACGTTGGATGGATGATGCAAAATGCTCACC	tTCCGCATCATGGGATACAA	C
W4	6-97	ACGTTGGATGCTGGCCATTAGCACACATTG	ACGTTGGATGTATGATGAGGATGGCACAGC	gACCGTCTCCTTTAAGTCTGC	G
W4	7-46	ACGTTGGATGTCGGCCTATCTTTGTCTGTG	ACGTTGGATGATCTCGAGTCGTTTGTGAGC	acGGGAGCCGTGAGCACGTTC	G
W4	11-128	ACGTTGGATGAGGACAGCCTGGAGCTGCG	ACGTTGGATGACACTTTCCGGTGTCCGTTG	aacGCGGGAGCTGGGGCTGCA	G
W4	7-34	ACGTTGGATGACAGGGTGGACACATCATC	ACGTTGGATGTTGTAGGTAAGGATGCCTCG	tgcCACATCATCTATAAGGCCG	C
W4	12-70	ACGTTGGATGGCCGTATGCCTCATGTTTTG	ACGTTGGATGAGCTAATTCTTGCGTGAGTG	tTCAACCAAGCATAAACGTGTC	A
W4	12-241	ACGTTGGATGCCCACAAACTCTTCATTGCC	ACGTTGGATGCCAGGCATGAAGCAAAGAAG	gagTCATTGCCTTGAAGGTTAA	G
W4	5-90	ACGTTGGATGCGCCAAACATGGTCATTTTTT	ACGTTGGATGCGCACAAATGGAATCTATTTT	cTTCTCATACATTTTCCTGTTTT	C
W4	3-215	ACGTTGGATGTGCCAGGTTGAAGAAGAACG	ACGTTGGATGTGCAACCTGCTGGTCTTGTG	gTGGCGTCGCTCTTTTTGCGGG	G
W4	8-67	ACGTTGGATGATACTCTCTATAGCAGTCGC	ACGTTGGATGCAGAGTGTAATAGAGACTCC	cCTCTCTATAGCAGTCGCTATAAA	C
W4	21-177	ACGTTGGATGGGAGGAAGTTCTCTCCGATG	ACGTTGGATGATGTCCCTGGGAACATTGAG	tggAGTTCTCTCCGATGTTGGATC	A
W4	10-121	ACGTTGGATGACACATCTCTGAACCAAGGG	ACGTTGGATGTCAGACATCCTACACGCAAC	agAAATCAGGGAAAAATACGATGT	G
W4	1-8	ACGTTGGATGTCAGTTGCTTACTCTTCACC	ACGTTGGATGTGCTGGTCGGTAGAAATTCC	aTTCACCATAACCACTTCAGCTGTG	C
W4	6-100	ACGTTGGATGTTTCGGAGGAACCGCAGTC	ACGTTGGATGCTTTAAACACGCACACGCAC	gTCGGAGGAACCGCAGTCGCTTCG	G
W4	19-166	ACGTTGGATGGATGACTACATAGCGTCACC	ACGTTGGATGACATCATGCTGGACAGCAAG	aAGGGAATCGTACCTATGTGGATGA	G
W4	8-59	ACGTTGGATGCCTGGATACAACATGGCAAG	ACGTTGGATGCGGTGAAGTCCTTGAAGTCC	aaCTAAATAAACTTTCCCTGACAATT	C
W4	12-251	ACGTTGGATGGAGGGAATGGCTTAAACCTG	ACGTTGGATGCTAGTAGGTTAGGTTCACAG	gGTTTTTTCCCTTCGTGTAAACGGGG	C
W4	13-139	ACGTTGGATGTCTTGACTGGTGAACATTTG	ACGTTGGATGCCAGAGCACATGTTAGTACA	gAAATTAGCTTTAGCATTATATGTAA	T
W4	16-153	ACGTTGGATGCGCAAAGCGGATATAAGAAC	ACGTTGGATGCAGCTCTCTGACTTTTATCG	cACGATAATGCCATAATTATCTTCTAT	T
W5	9-195	ACGTTGGATGTGGGTCACAACATAACGTGC	ACGTTGGATGTTTGTGTCACGTTAAGAGGG	CATCCATGGTTGCATTG	G
W5	9-110	ACGTTGGATGGTGTGCGTGTTTGTGTGTAG	ACGTTGGATGGCTTGATCCCAGCGTTCTCT	ACGCTGGCAGAGCAGAG	A
W5	2-26	ACGTTGGATGTCATGTAGCAGCAGGTGGTC	ACGTTGGATGAAGAAGAGAGCGTCGGAACAG	TGGGCAGCGGGTGTGAG	C
W5	14-140	ACGTTGGATGGGTACAATTACACATTGCCC	ACGTTGGATGTGTAGCGCCAGTTATCGTTG	GCCCTAAAAAGCCATTTG	G
W5	16-152	ACGTTGGATGTTACAGATCACAGCGAGGAG	ACGTTGGATGATGGGAGGCTGCGCATTAAG	aAGTGGGGCCTTAGGAGA	G
W5	4-218	ACGTTGGATGTGGCTATGTATCTAATTCC	ACGTTGGATGCGATACTTCCAATGGAAAGG	gCTCGTAGGGAAAGGAGA	A
W5	12-73	ACGTTGGATGTTGCCCACATGGTATGGAAG	ACGTTGGATGTGGTCCTGCGTTTCTTTTCC	ATGTGCTAATCTTGGTGTC	C
W5	20-170	ACGTTGGATGCCATGGCTTTTTCCACGTCC	ACGTTGGATGTGTATGTGTTGTGTCGCCAG	tcTTCCACGTCCTTCGCTGA	C
W5	7-40	ACGTTGGATGTTGAACACACTGTGTCAGGG	ACGTTGGATGTCACCACATGGTGGCAATAC	GCTGCCCCTGACTTAAGGGA	G
W5	20-220	ACGTTGGATGTTAAATCCTCCCTCCAGCTC	ACGTTGGATGGAGAACAATCCATAGAACGAG	gGGAGACCTGCAAAAAGTTA	C
W5	12-238	ACGTTGGATGTGCTGTAACGGATCTGAGAG	ACGTTGGATGTCGCAGGTACATTCGTAATG	TTGGATTATTAGCCTCGTCAT	G
W5	3-78	ACGTTGGATGATTCACTTTGTGTTCCTGGC	ACGTTGGATGTAGAGCCCTGAGATGTGTTG	cttcGTGTTCCTGGCTGGCGT	A
W5	4-189	ACGTTGGATGGGGTGCCCACACTTTTTCGT	ACGTTGGATGTCATCAGAGTATGTACTCGG	ccctACACTTTTTCGTCCAGGT	G

W5	15-146	ACGTTGGATGGTTTCAAATGAGGAGCCAGC	ACGTTGGATGATCCCGAAAGTGTCACAAAG	aggcGCCAGCTGGATAAAGTCT	C
W5	4-228	ACGTTGGATGCACTGAAAGAAAGAGACGGG	ACGTTGGATGTTCACCAGTGGTGGACACTC	ccctGCCCCCCCCAGCAGATGGT	C
W5	11-199	ACGTTGGATGTTCTGACACAATGCTTCTAC	ACGTTGGATGAGGCTACATGTTGCGCTCTG	TGACAACCACTTTAGATATGTCT	C
W5	7-57	ACGTTGGATGGCAGGCATGTTTGTGAACAG	ACGTTGGATGATCTGCGTACAATTGCCGAG	acgagCGTGTACGACTCGTACTG	C
W5	4-88	ACGTTGGATGAGGCATGTAGGAGTAGAAGG	ACGTTGGATGTCCATGCCCATCTACACCCT	ggAGGAGTAGAAGGCGTCGTTGC	C
W5	8-62	ACGTTGGATGCACTGTTCTCTACACAAAGA	ACGTTGGATGGGACCCAAATGTATTTCTGT	ctcgCTACACAAAGAACAGAGAAT	T
W5	9-109	ACGTTGGATGTTGCACCTGATGCTAGTCTG	ACGTTGGATGCCTAAGAAAGGTCTTACCAC	gacgGATGCTAGTCTGAGCTCGGT	C
W5	3-82	ACGTTGGATGAGGCCTCTGCATTTGAATGG	ACGTTGGATGGGAAGGAAGAGAGGAGTTAG	ccttGCCTTTTGCATGTCTGCTTGT	C
W5	8-64	ACGTTGGATGTCTACCAGATGATTGGTTCC	ACGTTGGATGCTCCCTTCTCTCAAGTAATG	ccacTGGTTCCTTATACTGTTTAGT	C
W5	2-18	ACGTTGGATGCCTTTCACTGACGGTGAATA	ACGTTGGATGTACTGTCCGAAAACGGTTCC	ggccCTGACGGTGAATAACTATGTA	C
W5	12-69	ACGTTGGATGGAACACATTAATGCTTCCTC	ACGTTGGATGAAGCAGATGATTATCACAGC	tcccTTAATGCTTCCTCATATCTTTT	C
W5	4-188	ACGTTGGATGACATAGAGTAATCATGAGG	ACGTTGGATGACATGCTAACCGTGTGTACC	ggTCATGAGGTTAATAAACCCTGTTA	C
W5	23-184	ACGTTGGATGAGTAACTCAATAAAGCGCTG	ACGTTGGATGGGACTATATCAACAGGTAGG	CGCTGTAACATGAACGATTAAGTATG	A
W5	2-25	ACGTTGGATGGTACCTCTTCCAAGTACTTT	ACGTTGGATGTATGTGCACATGCAGAACGG	ccaaCTCTTCCAAGTACTTTAAAAATC	G
W5	7-37	ACGTTGGATGCAGCGGTAAAAACAATGTCC	ACGTTGGATGCCACTGTTCGTCGATTTCGG	ccgCATCATAGTACCTATGGTTATATT	C
W6	10-120	ACGTTGGATGATCTCTAGCCCAGAGCCTGT	ACGTTGGATGTAGACCACGAGATGAAAGGG	GCCTCCTGTCTTGGCCC	G
W6	19-167	ACGTTGGATGATCAATCGGCCTGTTTGGTC	ACGTTGGATGGCTTTCGCAAAGACCACAAC	TTGGTCCGCTCTTCTGC	G
W6	18-160	ACGTTGGATGTCATGGACGTCCCGATGAAG	ACGTTGGATGACTACAGGTGTCTGAGAAGG	AGCGAGCTGTTTGTGGA	C
W6	15-236	ACGTTGGATGCAGGTGTGTGAGCTGCTGG	ACGTTGGATGTCCTACATGTGGAGGTCCTG	aTGCTGGCCACACAGAAC	C
W6	15-203	ACGTTGGATGAGAGCCAGCGTAACTGAAAC	ACGTTGGATGGAAAGAAAGTGCAGTGCAGG	cCAGGGAGACACGCTTGA	C
W6	1-3	ACGTTGGATGAAGTCCAAAAGGTTGGACAC	ACGTTGGATGTGTGTGTTTGTGTGTGCGTG	TTGGACACCCCTCCTCAAT	T
W6	7-44	ACGTTGGATGTGAGTTGGACATGAACCGAG	ACGTTGGATGCAGCCACATTTACACATGCC	TGAACCGAGTTTGTTGGCT	G
W6	10-116	ACGTTGGATGATCGTTAGCAAGGGATTCCG	ACGTTGGATGCTTCCCTGTGTCTGCAATTC	AAGTAAGTATTTGCTTGGC	T
W6	4-191	ACGTTGGATGTACATGCAGCCCCAGTCAAC	ACGTTGGATGGACGCAGCATGTTTCCTATC	CCCCAGTCAACAGGTGCCCA	G
W6	11-123	ACGTTGGATGTCTCTCAGGACTCGGTGAAC	ACGTTGGATGTGCTTCCTGATGAAGTCCTC	ccACAACGTGCTGCTGCCGC	A
W6	9-113	ACGTTGGATGATCGACTGAACGCTTGATGG	ACGTTGGATGCAGGATTCCAATCCACTCTG	gggAGACGAGTGAACTGACC	C
W6	14-143	ACGTTGGATGCCGCCATGGAATAGAAATAC	ACGTTGGATGCAACAACCGCACTACTATAAC	AGAAATACACACCAAATACAA	G
W6	22-180	ACGTTGGATGGCTGGGATGTATTTAGATACG	ACGTTGGATGCACTGCACACCAATGATCTG	CGAACGTAGGTGTTAAAAAAA	C
W6	7-42	ACGTTGGATGCTGGAACGCATTATCCTCTG	ACGTTGGATGTAGGGATAAACCATCAGGGC	gggaTACCAGGGAGATTTGGA	G
W6	1-12	ACGTTGGATGGGGACAACACAAATTCTCTG	ACGTTGGATGCCTCAGGTTTTTTGGATCAC	AACCTTATCACTCTGAAAATGA	C
W6	18-246	ACGTTGGATGAATCACAAGGCCACCCAGG	ACGTTGGATGTTAGCGTCGACTAATATTCC	cccaAGGCCCCCGCTCGTCCGTT	G
W6	23-212	ACGTTGGATGGGCAGGGATCAGTGACATAG	ACGTTGGATGCTAGTTTTTCTGCAGCAAAGC	TCTTTGAAAAACAAACTACTGAT	G
W6	16-150	ACGTTGGATGCAGAACAAATAGAAAACAGC	ACGTTGGATGTGGTTTGGTGGAACAAGAGG	ttCAAATAGAAAACAGCTTCAAT	A
W6	12-76	ACGTTGGATGTTCTTCACATGGTTCATGGG	ACGTTGGATGCCTTATAGGACGCTTTGCAC	gggAAGGTATCTCAGAGTCTTTG	C
W6	7-41	ACGTTGGATGCCATCAAGAAATAGCCTAAAG	ACGTTGGATGATCCCCGGCTTTGTTTTCTG	ggggTAGCCTAAAGGATTAACTGC	T
W6	2-14	ACGTTGGATGTAGACATGTACCCCGACTTC	ACGTTGGATGGTGGAACTCACATCTCTCAG	taccTGTACCCCGACTTCTCCGACG	G
W6	6-98	ACGTTGGATGTCAGAAGGACCTTAACCCTC	ACGTTGGATGGCCATTTAGAACCATTTGCAG	cctcAGGACCTTAACCCTCTAAAGT	G
W6	22-181	ACGTTGGATGCGTCATTCTATTGGACTCGG	ACGTTGGATGATCTTCTATGGGAAACAACG	GACTCGGATACATAGATATATACCG	G
W6	7-193	ACGTTGGATGGGTATGCTACGAGGTTAATG	ACGTTGGATGATGAAGGCCCCCTACTACAG	tAAACATGATACAGAATTAAGTTAC	T
W6	21-209	ACGTTGGATGGACCTCAGTAGGGAGGAATC	ACGTTGGATGCCCAAACTGTACCATGCAAC	gggAGAATACAACAAAACATCAAATG	G
W6	19-165	ACGTTGGATGATGTCAACTGATAACGCAAG	ACGTTGGATGCCCAGTCATTTTTTTTGAG	ggAATTAATGATATTTTCAGGGATTT	C
W6	18-207	ACGTTGGATGGTATATTTGCATTTATGCTC	ACGTTGGATGTCTGGACACATTGAAACTGC	ccTTATGCTCTTAAATTTTCTCTGTAC	A
W6	22-183	ACGTTGGATGTGACGGTCACCTGGTGGTC	ACGTTGGATGAGCTTATCAGAGCCCAGCAG	cctaaGGTCACCTGGTGGTCACCTGCC	A

Supplementary Figure 1 Primer sequences for all SNP assays.

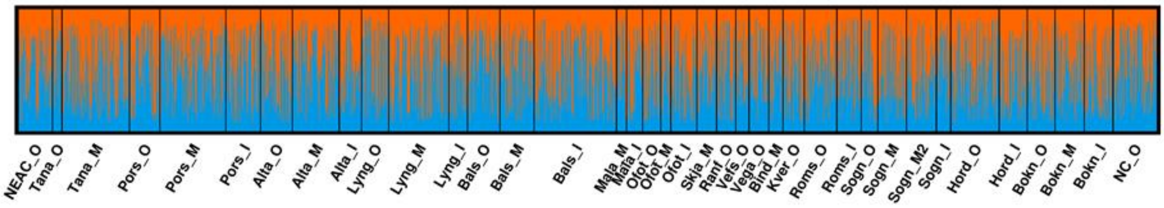

Supplementary Figure 2 STRUCTURE plot of Neutral markers with K=2aA)

A) 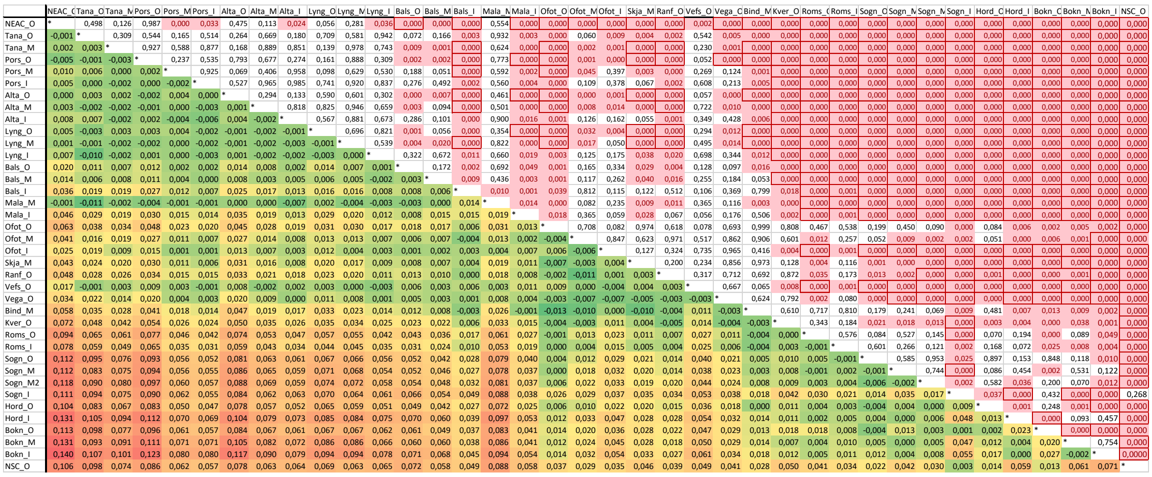

B) 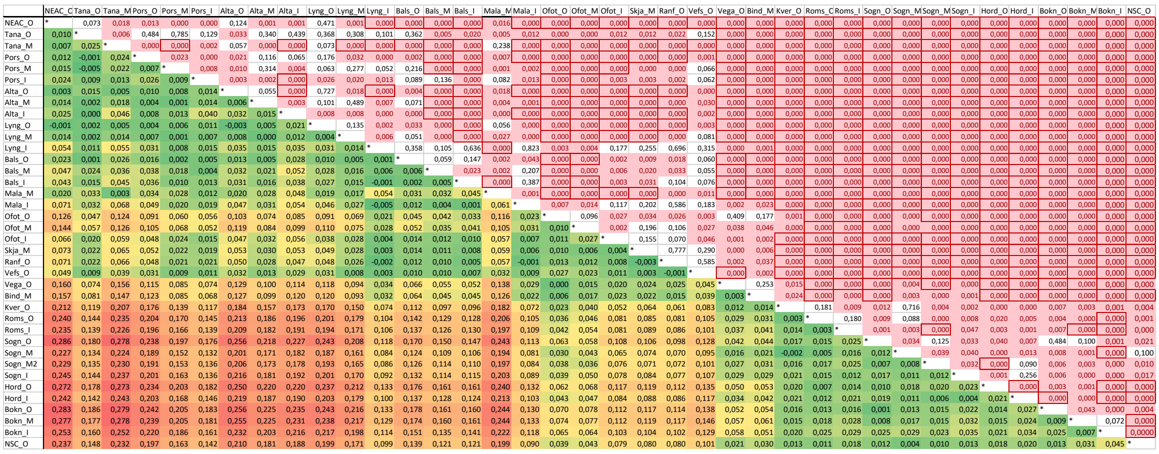

C) 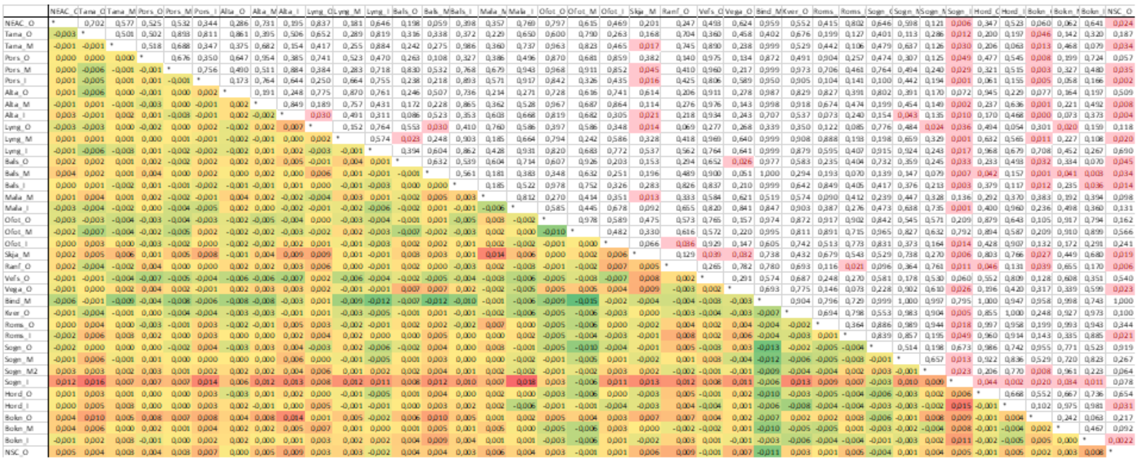

Supplementary Figure 3 Pairwise F_{ST} values. a) gradient markers, b) inversions and c) neutral. Pairwise F_{ST} values below diagonal, and P- values above diagonal. Pink windows give P-values below 0.05, and windows with red borders are found to be significant through Bonferroni corrections.

Grad_I	NEAC	Pors	Alta	Lyng	Bals	Ofot	Roms	Sogn	Hord	Bokn	NC
NEAC	*	0,1253	0,0549	0,2105	0	0,0019	0	0	0	0	0
Pors	0,0032	*	0,9674	0,579	0,0234	0,5489	0,0001	0,0043	0	0	0
Alta	0,0064	-0,005	*	0,7192	0,0024	0,3011	0,0005	0,0032	0	0	0
Lyng	0,0026	-0,001	-0,003	*	0,0372	0,3428	0,0002	0,0006	0	0	0
Bals	0,0231	0,0047	0,0106	0,0063	*	0,2672	0,0163	0	0	0	0
Ofot	0,0136	-8E-04	0,0017	0,001	0,0015	*	0,003	0,0043	0	0	0
Roms	0,0495	0,0179	0,0203	0,0252	0,0065	0,0139	*	0,0047	0,0352	0,0094	0
Sogn	0,0443	0,0153	0,0193	0,0255	0,0282	0,0168	0,0184	*	0	0	0,1684
Hord	0,1036	0,056	0,0588	0,0667	0,0378	0,0448	0,0075	0,0515	*	0,4004	0
Bokn	0,1045	0,0605	0,0572	0,0583	0,0395	0,0458	0,0103	0,0516	0,0004	*	0
NC	0,0474	0,0261	0,0305	0,0395	0,0357	0,0192	0,0354	0,0042	0,0666	0,0725	*

Inv_I	NEAC	Pors	Alta	Lyng	Bals	Ofot	Roms	Sogn	Hord	Bokn	NC
NEAC	*	0	0,0004	0	0	0	0	0	0	0	0
Pors	0,0332	*	0	0,0401	0,0036	0,0017	0	0	0	0	0
Alta	0,0234	0,0337	*	0,0002	0	0	0	0	0	0	0
Lyng	0,0586	0,0103	0,03	*	0,7255	0,0318	0	0	0	0	0
Bals	0,0457	0,0098	0,0321	-0,0019	*	0,0008	0	0	0	0	0
Ofot	0,0887	0,0179	0,0613	0,011	0,0164	*	0	0	0	0	0
Roms	0,2632	0,1479	0,2052	0,122	0,1455	0,0773	*	0,0494	0,0007	0,0001	0,0004
Sogn	0,2636	0,1322	0,1947	0,0986	0,1198	0,0642	0,012	*	0,2045	0,0002	0,0297
Hord	0,2674	0,1492	0,2011	0,1124	0,1381	0,0787	0,0207	0,0043	*	0	0,0004
Bokn	0,2866	0,1716	0,2315	0,1338	0,1635	0,1011	0,0262	0,0339	0,0266	*	0
NC	0,2599	0,1483	0,2002	0,1152	0,1384	0,076	0,0165	0,0108	0,0155	0,0373	*

Grad_O	NEAC	Pors	Alta	Lyng	Bals	Ofot	Roms	Sogn	Hord	Bokn	NC
NEAC	*	0,9937	0,3542	0,0178	0	0	0	0	0	0	0
Pors	-0,006	*	0,6292	0,0525	0,0018	0	0	0	0	0	0
Alta	0,0006	-0,001	*	0,5811	0,001	0	0	0	0	0	0
Lyng	0,0075	0,0058	-9E-04	*	0,0008	0	0	0	0	0	0
Bals	0,0189	0,012	0,0119	0,0139	*	0,0006	0	0	0	0	0
Ofot	0,0632	0,0497	0,0444	0,0318	0,0181	*	0,5993	0,166	0,0581	0,0015	0
Roms	0,0978	0,0797	0,0746	0,0582	0,0414	-0,002	*	0,1228	0,0482	0,0001	0
Sogn	0,115	0,0951	0,0819	0,0689	0,0522	0,0052	0,0043	*	0,7325	0,8222	0,0001
Hord	0,1097	0,0884	0,08	0,0661	0,0498	0,0065	0,0043	-0,002	*	0,2405	0
Bokn	0,1169	0,0997	0,0843	0,0684	0,0616	0,0162	0,0159	-0,004	0,0021	*	0,0018
NC	0,1097	0,0897	0,079	0,0696	0,0708	0,0364	0,0415	0,0221	0,0143	0,0108	*

Inv_O	NEAC	Pors	Alta	Lyng	Bals	Ofot	Roms	Sogn	Hord	Bokn	NC
NEAC	*	0,0127	0,1257	0,4748	0,0001	0	0	0	0	0	0
Pors	0,0118	*	0,0213	0,1817	0,0028	0	0	0	0	0	0
Alta	0,0034	0,0102	*	0,7252	0,0038	0	0	0	0	0	0
Lyng	-0,0006	0,0043	-0,0027	*	0,0309	0	0	0	0	0	0
Bals	0,0232	0,0159	0,0133	0,0099	*	0	0	0	0	0	0
Ofot	0,1256	0,0912	0,1025	0,0913	0,0452	*	0	0	0	0	0
Roms	0,2404	0,2042	0,2134	0,2013	0,1418	0,0361	*	0,0065	0,0222	0,0046	0,0012
Sogn	0,2861	0,2377	0,2556	0,2432	0,1699	0,0626	0,0149	*	0,0398	0,4801	0,0212
Hord	0,2724	0,2335	0,2501	0,2371	0,1759	0,0615	0,0069	0,0103	*	0,003	0,0001
Bokn	0,2828	0,2422	0,2556	0,2429	0,1779	0,0703	0,0133	0,0009	0,0139	*	0,0036
NC	0,2366	0,1971	0,2097	0,1992	0,1392	0,0394	0,0112	0,0116	0,0183	0,0127	*

Supplementary Figure 4. The pairwise F_{ST} values on the Inner/Outer dataset. Pairwise F_{ST} values to the bottom left half, and P- Values at the top right. Pink windows give P-values below 0.05, and windows with red borders are found to be significant through Bonferroni corrections.
